# Artificial intelligence in human resource management: models for recruitment, training, performance, compensation, and retention

**DOI:** 10.3389/frai.2026.1718244

**Published:** 2026-02-06

**Authors:** Meysam Safshekan, Ardalan Feili, Ali Shojaeifard, Shahryar Sorooshian

**Affiliations:** 1 Department of Management, Apadana Institute of Higher Education, Shiraz, Iran; 2 Department of Management, Payam Noor University, Tehran, Iran; 3 Department of Business Administration, University of Gothenburg, Gothenburg, Sweden; 4 Faculty of Engineering and Sustainable Development, University of Gävle, Gävle, Sweden

**Keywords:** artificial intelligence, business management, compensation, human resource management, performance, recruitment, retention, training

## Abstract

**Introduction:**

In the era of rapid technological advancement, artificial intelligence (AI) has emerged as a transformative force across various industries, including human resource management (HRM). This study examines the application of AI in HRM systems, with a focus on recruitment, hiring, training, performance management, compensation, and human capital retention.

**Methods:**

This study adopts a qualitative research approach. Experts in artificial intelligence and human resource management were identified, and data were collected through qualitative methods. The data were analyzed using thematic analysis.

**Results:**

The findings reveal the introduction of AI application models across various HRM systems. These models demonstrate how AI enhances efficiency and effectiveness in key HR functions.

**Discussion:**

The results highlight the transformative potential of AI in HRM by enabling data-driven decision-making and improving workforce planning. This research provides valuable insights for human resource professionals seeking to leverage AI to enhance organizational performance across industries.

## 1 Introduction

Artificial intelligence (AI), a highly modern, efficient, and popular digital tool, has become a cornerstone for operations in organizations worldwide by increasing efficiency and reducing human errors. One such operation is human resource management (HRM), a ubiquitous process in any organization. AI is revolutionizing numerous industries, and HRM is no exception. With recent technological advancements, adopting new trends has become imperative for companies. AI is considered one of the newest technologies (Harari, 2017). However, the discussion surrounding human cognitive bias in using AI for predicting sales or company outcomes has heated up (Teleaba et al., 2021). AI is not infallible, and input data errors can lead to biased output (Huang and Rust, 2021). Nevertheless, there is a growing concern about the extent to which AI should be used, a group of professionals view AI as a threat (Jaworek and Stuss, 2025; Mirbabaie et al., 2022). In this regard, it emphasizes the possibility that AI could replace all human capacities, including emotions and imaginative power (Harari, 2017). In this context, considering HRM and AI, examining the extent of organizations’ AI usage is unavoidable. Apart from using AI in the recruitment process, AI can be beneficial for other HR practices like performance management, retention, training, and development (Wislow, 2017).

Transparency is a vital factor in AI deep learning systems (Sharma et al., 2021). Data transparency categorizes privacy, while humans train machine learning models through an artificial neural network where decisions are not justified correctly, facing black box problems (Selbst and Barocas, 2018). These black-box problems pose a risk to companies as deploying algorithms is not easily explainable, creating biases in the AI system (Roselli et al., 2019). Employees who perceive AI systems as competitors of company resources oppose its acceptance (Choudhury et al., 2020). The emergence of AI-based systems in business organizations will significantly alter the workforce demographics, the nature and meaningfulness of jobs, the employer-employee relationship, the human-technology relationship, customer experience, and competitive advantage in the dynamic market environment (Wilson et al., 2017). By entering such an industrial revolution, organizations are gradually adopting emerging technologies like AI, machine learning, and big data, which can not only perform previous physical tasks but may even replicate high-level cognitive abilities that utilize continuous learning processes (Gibbons, 2019; Walsh, 2018).

However, some routine tasks in HRM, such as recruitment, must be performed by HR personnel from time to time. Moreover, this scenario is changing today due to technological advancements. Using AI can help HR managers make quicker decisions and streamline processes. In this regard, many philosophers have interpreted the concept of AI in different ways (Upadhyay and Khandelwal, 2018). In reference, Russell and Norvig (1995) described a conceptual view of AI as a modern approach that presented two dimensions of AI. One dimension is human-based, where the system thinks and acts like a human. The other dimension is rationality-based, where systems act and think rationally. According to Winston (1992), AI refers to the perception, reasoning, and action abilities exhibited by individuals. Additionally, Winston stated that intelligent humans and intelligent machines have complementary strengths, where humans and computers leverage capabilities that cannot be achieved by themselves (Winston, 1992; Forskning, 2017). At this stage of technological advancement, many companies are currently moving towards AI to accomplish tasks with greater efficiency within a timeframe. This helps reduce workforce stress and guides the utilization of resources that are intelligently implemented to achieve high business performance (Tiwari, 2020). However, such thinking is primarily done by creating the usual pessimistic instinct of humans (Rosling et al., 2018). Therefore, examining the positive aspects of using AI in jobs is necessary.

Human capital is a distinguishing element of an organization as it is an intangible resource that is difficult for competitors to imitate, thus giving a potential competitive advantage to any organization (Kearney and Meynhardt, 2016). Despite the fact that AI has been rapidly integrated into organizational procedures, its use in HRM remains inconsistent and unclear (Lutfi and Mohammadi, 2025; Azhar and Imran, 2024). Until now, majority of research efforts focused on specific HR functions, like training (e.g., Maity, 2019) or recruitment (e.g., Alrakhawi et al., 2024). Multimedia systems, gamification, and generative AI can empower individuals to set up virtual training sessions (Khan et al., 2025). There is a scarcity of research that systematically models the application of AI across all HRM practices (Mendy et al., 2025; Chhabra and Malhotra, 2024; Budhwar et al., 2022). This lack of a thorough understanding creates uncertainty for human resource professionals and organizations attempting to implement AI responsibly and effectively. Yet, the fact is that AI may soon replace humans in many instances (Marr, 2020).

AI’s role in an organization is to improve the efficiency and effectiveness of HR performance by streamlining and refining various management processes (Nankervis et al., 2021). For HRM, AI enables understanding and controlling the data collection process to incorporate this process into organizational and economic efficiency strategy (Varma et al., 2023). In an organization that uses AI, various areas where HRM operates can be addressed, including talent acquisition and recruitment, training and development, performance analysis, career development, compensation, and employee relocation (Abdeldayem and Aldulaimi, 2020; Qamar et al., 2021; Yahia et al., 2021). In this paper, we aim to examine AI applications in HRM and propose a comprehensive (multi-approach) implementation model.

## 2 Literature review

This section gives an overview of two concepts, HRM and AI, and then goes on to discuss how they are integrated.

### 2.1 Human resource management

Researchers have provided numerous definitions of HRM, ranging from simple and concise to more comprehensive ones. HRM has been defined as “the process of managing human talent to achieve organizational goals” (Abdullah, 2009). This suggests that the process of man-aging human talent encompasses recruitment and selection, rewards and benefits, industrial and labor relations, as well as occupational safety and health management in organizations (Abdullah, 2009). The following definitions, presented in Table 1, can help understand the term HRM. They are selected from the first definition of HRM while considering the component definitions of the functions of HRM and our study.

**Table 1:** Existing definitions of HRM.

Source	Definition
Guest (1987)	HRM consists of a set of policies designed to maximize organizational integration, employee commitment, flexibility, and quality of work.
Schemerhorn (2001)	HRM means how you can acquire and develop a talented workforce to assist the company in achieving its goals as well as its various missions, visions, and objectives.
Armstrong (2006)	“A strategic and coherent approach to the management of an organization’s most valuable assets – the people who work there and who individually and collectively contribute to the achievement of the business objectives.”
Boxall and Purcell (2011)	HRM refers to all activities concerned with the management of work and people in organizations.

The HRM System consists of four main elements:

- Recruitment and selection: includes the process of recruiting and recruiting human resources with appropriate skills and competencies for the organization.
- Training and development: includes training and development processes for employees to improve skills and improve their performance.
- Performance evaluation: involves evaluating and measuring employee performance using concrete criteria and providing appropriate feedback.
- Reward management and retention: includes reward management policies and procedures, promotion and retention of employees in order to encourage them to perform better and retain them within the organization (Dessler, 2002).

The history of HRM can be traced back to the early 1800s during the era of industrialization and apprenticeship. It developed further with the advent of the Industrial Revolution in the late 1800s. Frederick Taylor, in the 19th century, suggested that an introduction should be made with a combination of scientific management and industrial psychology. By doing so, workers would not only be managed from the perspective of their jobs and performance but also their psychological well-being would be maximized. Moreover, the widespread changes in technology, organizational growth, and increasing concerns of unions and the government led to the development of the personnel office in the 1920s. Additionally, some researchers argued that HRM is said to have originated from the term “personnel management.” Historically, the term “PM” emerged from World War II in 1945. It was an approach used by personnel specialists to differentiate them from other managerial tasks by professionalizing personnel functions in the managerial domain. The traditional tasks of PM were “hiring or firing,” in other words, “recruiting and terminating” personnel, setting aside other tasks such as compensation and training. However, many critics and concerns expressed the vague goal of the PM role in HRM. In this regard, the term HRM gradually replaced the term PM (Lloyd and Rawlinson, 1992). With the evolution and changes in the world of business management, the renaming of PM to HRM seems more appropriate to fit many of the natures, concepts, and philosophies of human resources (Noon, 1992; Armstrong, 2000). It has been argued that there seems to be a small difference between PM and HRM with a different label (Ahammad, 2017). There is a very strong and valid relationship between technology and humans, which is needed as a benchmark to support business goals and objectives. Therefore, technology can serve as a tool to support and develop management and decision-making systems that aid in the reconstruction of organizational modes (Jatoba et al., 2019).

### 2.2 Artificial intelligence

AI plays a major role in this research. There is a growing use of AI and several applications that are currently being used in various industries. It is important to have an overview of AI to understand how AI is used. Generally, AI can be defined as algorithms designed to mimic human behavior and thought (Chenik, 2018). Human intelligence has played a significant role in human development by understanding, implementing, and expanding their expertise (Thomas, 2017). Human intelligence helps a lot, people can now drive from city to city, migrate effortlessly from one continent to another, and connect to the whole world through smartphones and the internet where technological growth is very high (Christakis, 2010). Salomon et al. (1991), previously showed in their scientific research that human intelligence not only makes the planet more technologically complex, but machines are also intelligent, which makes people smarter. The most obvious scenario is that computers provide additional memory for people to store data in Witten et al. (2016). However, computer implementations of intelligence, including algebra, logic, philosophy, linguistics, and decision theory, are much more complex (Maini and Sabri, 2017). This technological advancement of AI is one of the most important areas of science and engineering that can perform human tasks more efficiently (Russell and Norvig, 1995). Moreover, data processing, machine learning, and AI have recently become increasingly popular and have changed the game in terms of data and computation (Maini and Sabri, 2017). As technical devices such as robots or computer systems increasingly replace roles previously performed by humans, it is now necessary to understand how machines think and how this affects our future lives (Maini and Sabri, 2017). For example, in 2015, Google trained an AI agent capable of answering questions, discussing ethics, and making statements (Maini and Sabri, 2017). An example where AI has previously been used in the health sector is the rapid scaling of irregular findings from cancer patient medical diagnoses (Maini and Sabri, 2017). While some depict the concept of AI as something far in the future, it is a centuries-old idea that has advanced significantly (Press, 2016). Alan Turing made this speech at the London Mathematical Society and stated that what we want is a computer that can learn from experience (Press, 2016).

In 1956, a group of mathematicians and scientists at Dartmouth College provided a definition that they called AI and there is an independent “language” that comes with AI (Vella, 2017). In general, AI defines something that enables machines to act like humans, which has two defining characteristics: the ability to mimic human intelligence and the endless ability to learn (Raub, 2018). AI refers to techniques that use statistical techniques to help machine learning so that machines can become better at specific tasks (Raub, 2018). As far as definitions are concerned, there are several different types of AI, but the idea can be expressed in a few words, these systems can think and learn (Russell and Norvig, 1995). AI technologies are used in dynamic and increasingly specialized settings (Davenport and Kirby, 2016). Post-automation economies are now occurring due to improved smart technologies, which are rapidly eliminating informal jobs in different places (Brynjolfsson and McAfee, 2014). As AI implementation is increasing, businesses are facing difficult questions about the impact of AI on their work. It is argued that “a computer scientist may now be trying to create an algorithm for every ability you can think of” (MacCrory et al., 2014). In these respects, AI and other smart technologies are often seen as the focus of an unprecedented wave of automation. They are seen to be shifting the decision-making process towards the cognitive and informational (Kelly, 2012; MacCrory et al., 2014). Managers of leading American companies have ranked AI and machine learning as major business advancements in the future market (Gutierrez, 2017). Today, Gartner innovations are essentially all AI-related (Panetta, 2017). The recent boom in AI has created perspectives on the potential impact on the human race, with some believing it could herald a frightening future dominated by machines (Bini, 2018). In contrast, others believe that job creation will be significantly higher due to improved decision-making through the use of AI.

On this note, the question arises of what exactly AI means. Different definitions of AI have been provided by different researchers. AI does not have a fixed description and depending on the conditions under which it is placed, the program for which it is used, and its intelligence are subject to different interpretations (Jarrahi, 2018). In general, AI can be considered a machine that simulates general human skills such as understanding, speaking, and solving problems, which makes it act like an intelligent individual (Jarrahi, 2018; Russell and Norvig, 1995). The following definitions, presented in Table 2, can help understand the term AI. They are selected from the first definition of AI provided by different researchers and based on the above discussion, these definitions help to better understand the concept of AI.

**Table 2:** Existing definitions of AI.

Source	Definition
Turing (1950)	Turing equated “intelligence” with a human’s ability to engage in any cognitive task.
Simmons and Chappell (1988)	AI refers to behavior exhibited by a machine that, if exhibited by a human, would be considered intelligent.
Winston (1992)	AI is the capacity to understand, reason, and act.
Poole et al. (1998)	AI is the study of agents - intelligent agents - that understand their environment and take actions to maximize their chance of success at some task.
Russell and Norvig (2016)	AI as systems that mimic cognitive functions, often including human-like traits such as learning, reasoning, and problem solving.
Kaplan and Haenlein (2019)	AI can be described as a system that acquires information from its environment and uses it to achieve specific goals through adapting to the situation, learning, and reasoning.
Van Esch et al. (2019)	Any intelligent agent (e.g., a device) that differentiates between different environments and can take actions to increase success in achieving predefined goals.
Malik et al. (2020)	AI in business refers to the development of intelligent machines or computer systems that can learn, react, and perform activities like humans for a wide range of tasks.
Makarius et al. (2020)	The ability of a system to correctly interpret external data, learn from such data, and use such learning to achieve specific goals and tasks through flexible adaptation.
Dwivedi et al. (2021)	Increasing the ability of machines to perform roles and tasks that are currently performed by humans in the workplace and society in general.

The features of using machines that are often implemented to enhance intelligence include: natural language processing, machine learning, and computer vision (Jarrahi, 2018). Emerging and developing technologies that are increasingly being utilized by HRM professionals highlight the need for a critical understanding of the involved processes, as the benefits for organizations and employees may be undermined without a clear roadmap for future integration (Barro and Davenport, 2019). Machine learning and deep learning are key processes in the overall AI technology used in HRM. The roots of machine learning and deep learning lie in pattern recognition and in the concept that algorithms can learn from recorded data without being programmed to do so (Rodgers, 2020). Machine learning helps systems to learn from them. Collecting and organizing data (Jarrahi, 2018). Deep learning is a branch of machine learning that teaches computers to learn from large amounts of data through neural network architecture.

This is a more advanced form of machine learning that breaks down data into abstract layers. Instead of organizing data for execution in predefined equations, deep learning adjusts fundamental parameters regarding the data and trains the computer to learn independently by identifying patterns using multiple layers of neural networks for processing (similar to the neurons in the brain) (Rodgers, 2020). After sufficient training, deep learning algorithms can predict or interpret highly complex data with minimal human supervision, such as in financial trading (Barro and Davenport, 2019). Natural language processing enables systems to understand and recognize how humans communicate (Jarrahi, 2018). NLP refers to the ability of machines to communicate effectively with humans in their native language. This capability enables machines to better understand both speech and text and to generate relevant responses to stimuli they receive from humans. Such AI methods have become more common in interactions with customers and assistance to employees, as chatbots and language analysis algorithms have begun to introduce themselves, simplifying various processes in human resource systems such as employee onboarding, recruitment, training, and leave requests (Garg et al., 2021; Majumder and Mondal, 2021). A chat robot can interact with a person and independently answer their questions (Eubanks, 2018; Majumder and Mondal, 2021; Shum et al., 2018). AI can be traced back to 1950, when Alan Turing, an English mathematician, devised a test to see if a machine could mimic human cognitive functions to recognize patterns (Batra et al., 2018). Then, in 1956, when John McCarthy invited academics and industry experts from interdisciplinary fields around the world to discuss the importance of computers that consume data and mimic human behavior, it gained even more popularity (Garg et al., 2021; Herath and Mittal, 2022). These data share and create the opportunities that have become available worldwide with the new advancements in higher computational processing power (Akter et al., 2020). These technological advancements impact companies and completely transform the market (Sharma et al., 2021).

Since the invention of high computational power in 1956, theories related to AI have been developed for several years (Cohen and Feigenbaum, 2014). Many specialists and experts in the industry have provided various definitions of AI as machines with human-like cognitive abilities (McGettigan, 2017), enabling them to manage complex situations (Malik et al., 2021). In addition, AI helps provide several solutions for decision-making in companies to assess analytical, intuitive, and empathetic intelligence (Bader and Kaiser, 2019; Kar et al., 2022). AI, which encompasses data, algorithms, and computations, has made significant progress in recent years (Messner, 2022). As a result, AI is machine learning that employs an algorithm to process raw data, capable of generating meaningful outputs through models (Sarle, 1994).

### 2.3 The integration of AI in HRM

AI technology is transforming every aspect of HRM. Currently, virtual assistants are helping in HRM activities such as recruitment, employee selection, training and development, performance evaluation, and employee engagement. The initial and vital stage of HRM is human resource planning. The human resources information system can help in this regard. HRIS is described as “a systematic technique for acquiring and maintaining the necessary data by an organization regarding the characteristics of the organizational unit, personnel activities, and human resource needs” (Dwivedi et al., 2021). HRIS can assist with human resource planning, job roles, performance reviews, training initiatives, and more (Chen and Li, 2019). AI technology is gradually transforming all aspects of HRM functions. Functions of HRM such as recruitment, selection, training and development, performance management, and employee engagement are all carried out with the assistance of virtual assistants. Human resource planning is the first and most important step in HRM. HRIS plays a vital role in this process. HRIS is described as a systematic method for collecting, storing, maintaining, retrieving, and validating the data required by an organization regarding human resources, personnel activities, and the characteristics of the organizational unit; it can also be useful in human resource planning, creating job descriptions, performance evaluation, designing training programs, and more (Kovach and Cathcart, 1999).

Mendy et al. (2025) in a study titled “Artificial Intelligence in the Workplace—Challenges, Opportunities, and Human Resource Management Framework: A Critical Review and Research Agenda for Change,” aimed to conduct a critical and systematic review of the literature related to artificial intelligence-human resource management in contemporary changing organizations. Using a meta-synthesis approach, they synthesized and critically reported on 47 articles. The results highlighted three important challenges and opportunities: job performance challenges, organizational and human resource performance challenges, and opportunities for collaborative intelligence in changing organizations.

Chowdhury et al. (2023) in their research titled “Unlocking the Value of AI in HRM through an AI Capability Framework,” aimed to systematically review the multidisciplinary literature arising from international business, information management, operations management, public management, and HRM. Their goal was to provide a comprehensive and objective understanding of the organizational resources needed to develop AI capabilities in HRM. They conducted a systematic literature review following the proposed protocol in the literature to ensure that the review process is transparent and easily replicable, while critically organizing research themes. The results showed that organizations need to look beyond technical resources and focus on developing non-technical aspects such as skills, human competencies, leadership, team coordination, organizational culture, innovation mindset, governance strategy, and AI employees. Ultimately, integrated strategies are necessary to benefit from the adoption of AI.

In a study by Jain et al. (2023) they sought to examine the literature and research methodologies about the use of AI in the human resources domain. Using secondary data from applied research, they talked about the benefits, drawbacks, and potential applications of this technology in the future. An outline of the use of AI in HRM operations has been given. The findings indicated that AI-assisted decision-making could free up human resources staff members to concentrate on critical duties. AI is being used more and more in human resources, assisting businesses in moving from reactive to proactive problem-solving and turning HR divisions into hubs for strategic decision-making. Numerous case studies demonstrate the benefits and drawbacks of this.

Agustono et al. (2023) conducted a study examining AI in HRM Practices. By using a review study that involved collecting data from various articles and scientific journals related to the discussed topics. The results of the literature review showed that the use of AI in HRM has the potential to enhance the efficiency and effectiveness of HRM processes and create added value for companies in improving their performance and competitiveness. ACF is used as a framework to assess a company’s ability to implement AI in HRM, taking into account policies, technological infrastructures, human resource capabilities, and organizational culture. It provides suggestions and recommendations for companies to develop AI capabilities in HRM, such as conducting training and development for human resources, strengthening technological infrastructure, and creating an organizational culture that supports the use of AI technology. In addition, several factors were identified that could impact the implementation of AI in human resources.

In a study titled “Intelligent Human Resources for AI Adoption: A Systematic Literature Review,” Jatobá et al. (2023) critically analyzed prior research, looking at clustering analysis methods and content analysis processes. The future of work, training and AI, recruiting and AI, and strategic human resources and AI are the four topic clusters that the author identified. Finally, it concludes that the ethical debate over data privacy, equality, openness, and dependability in the creation of human resource systems is essentially where the conversation about the nature of work in the future starts. It also highlights the future cluster of work, especially given that there will be a significant revolution in the way specialists in this field function, which will alter the way that existing operations in this sector are carried out.

## 3 Method

This research uses a qualitative data-driven approach. The choice of method for conducting qualitative research heavily depends on the researcher’s perspective and the research objective (Creswell and Poth, 2016; Lewis, 2015). The present research is applied in terms of its objective and qualitative in nature and method; since we are aiming to model the minds of experts, it is descriptive and cross-sectional in terms of time. Through the study of books, articles, existing documents, and records, the literature and background of the issue related to the research topic, which has been gathered in a library manner, in addition to this method, by visiting individuals or environments and establishing direct communication with people, institutions, among others. It has been collected in a field manner. The tool for gathering this research has been interviews, conducted verbally and through direct communication with the source of information, involving questions and answers to obtain data.

### 3.1 What is thematic analysis?

Thematic analysis is a method for identifying, analyzing, and determining patterns in existing qualitative data. Some of the most important features of this method include being a common, flexible, quickly learnable approach with a basic process that can be used in most qualitative methods, emphasizing text extraction and semantic modeling based on the interpretive paradigm, and focusing on immersion and review of data to shape main and sub-themes, ultimately leading to a theoretical model of all themes (Guest et al., 2012; Clarke and Braun, 2013). The main key concept in this method is the researcher’s perspective based on logical reasoning about a specific topic, the pattern or meaning derived from the collected data in a study, and statements or sentences used to identify the meaning of the data or provide an explanation resulting from the analysis of the coding (Guest et al., 2012). Given the importance of data analysis in thematic analysis research and the iterative actions taken throughout the data to identify themes, the collected data is reviewed and coded until the main themes are reached. The most important output in the data analysis phase is the identification of the main themes, which are the themes themselves. All collected data is standardized into text, and by assigning codes to common concepts, the groundwork is laid for theme development.

### 3.2 Sample selection

This research is based on qualitative research methods, and therefore the author used a non-probability sampling method. Additionally, for non-probability samples, purposive and snowball sampling are used (Bell et al., 2022). Purposive sampling, where study participants must have a complete understanding of the research topic (Neuman, 2014). Purposive and snowball sampling are other non-probability sampling methods (Bell et al., 2022). Additionally, purposive sampling is based on the assumption that the researcher intends to explore, understand, and gain insights, and therefore must select a sample from which the maximum amount of information can be gathered (Patton, 2002). Snowball sampling is a technique in which the researcher approaches relevant participants and then uses these contacts to access other participants (Bell et al., 2022). Consequently, this approach is referral-based. One of the drawbacks of this technique is that participants seem to nominate colleagues they know and who likely share similar characteristics. This will certainly not result in a diverse sample; however, this technique is suitable when the sample size is small and finding participants is difficult. In this study, however, purposive sampling was combined with network sampling or snowball sampling based on the obtained contacts. By combining these two approaches, a wider range of identifiable populations emerges and the interviews will be more resultful (Collis and Hussey, 2014). Since we want to explore AI concepts in the hiring process, our goal is to interview individuals who have knowledge of AI and human resource management. Therefore, we consulted with human resources departments of companies, human resources consultants, and professors of human resources management and artificial intelligence.

### 3.3 Data collection

The data collection tool for this research was interviews, which were conducted orally through direct contact with the information source and included questions and answers to obtain the data. In the present study, a semi-structured interview format was used. The semi-structured interview model was the primary model used, employing pre-designed questions. Additionally, the interviewer asked extra questions during the interviews. The questions raised in this study were based on a collection of literature and fields that the author believed were important for addressing the research question, which was approved by the supervisors and advisors. As mentioned earlier, in a semi-structured interview, questions are designed in advance, and during the interview, the interviewer can ask any questions that come to mind. Additionally, the interviewee is allowed to express their opinions that were not included in the interview questions or any points they feel are necessary to improve the research. The text of the questions is provided in Table 3.

**Table 3:** Interview questions.

Interview questions	
Part 1: general information	Please introduce yourself and describe your experience in the field of human resources.
Part 2: applications of artificial intelligence in various human resource management departments	To what extent do you think AI can be effective in attracting and hiring talented individuals?
	How do you think artificial intelligence can help improve employee skills and performance?
	To what extent do you think AI can be effective in providing accurate and fair feedback to employees?
	How do you think AI can help create a fair and transparent system for compensating services and benefits?
	How do you think artificial intelligence can help create a happy and vibrant work environment?
Part 3: additional questions	Please provide examples of AI tools or platforms used in the field of human resources.
	In your opinion, what skills will be necessary for human resources professionals in the future?
	What resources do you recommend for further study on AI in human resources?
	In your opinion, what role will artificial intelligence play in the future of human resource management?
	What are the main challenges and obstacles to the successful implementation of AI projects in the field of human resource management?

To conduct interviews with the experts in this study, an email in the format of an official letter was first sent to all participants to schedule interviews, and after obtaining their consent, the interviews were conducted. These interviews were conducted in the spring of 2025. The experts in this research are individuals with experience in artificial intelligence and human resource management who have authored several academic scientific articles and books related to this study, and some of whom have also had practical operational experience in organizations. Therefore, interviews were conducted with key individuals (such as human resources managers and expert professors in artificial intelligence). In Table 4, descriptive information about the participants is presented.

**Table 4:** Descriptive characteristics of participants.

Row	Gender	Age (years)	Educational qualification	Work experience	Number of published documents or completed Projects
1	Male	43	PhD in HRM	13 years as a consultant, instructor, and executive manager.	14
2	Male	55	PhD in HRM	25 years of teaching, executive management, and business consulting.	25
3	Male	56	PhD in HRM	25 years of teaching, management consulting, and human resource development.	33
4	Male	40	PhD in Computer Engineering - AI	18 years as CEO and HR manager.	4
5	Female	46	PhD in Organizational Behavior and Human Resources	22 years of teaching, executive work, and business consulting.	16
6	Female	52	PhD in HRM	16 years of teaching and management consulting.	31
7	Male	44	PhD in Computer Engineering - AI	5 years as the CEO of a private company regarding AI.	3
8	Male	40	PhD in HRM	10 years of teaching and management consulting.	6
9	Male	51	PhD in HRM	12 years of teaching and management consulting.	12
10	Male	49	PhD in Computer Engineering - AI	8 years as a Human Resources Manager.	5

This research complies with all ethical standards related to qualitative studies. Raising ethical considerations is crucial to ensure study quality and prevent unethical practices during research (Bryman and Bell, 2011). Like any research study, the authors must be aware of the ethical aspects related to the development of the research. Ethics relates to the values or moral principles that form the basis of the rules for conducting research (Collis and Hussey, 2014). As explained below, the most important ethical considerations in research are presented, and our arguments for adhering to these ethical guidelines are provided. Our goal is to follow these guidelines throughout our research. (1) Participants and individuals affected by the research must be respected, and trust must be established between them and the researchers, (2) it is the researchers’ responsibility to carefully assess the risk of harm to participants in the research, (3) the confidentiality and anonymity of respondents must be maintained throughout the research process, and (4) participants should not be lied to or deceived about the study (Saunders et al., 2009; Bryman and Bell, 2011). Therefore, before data collection, participants were provided with complete and transparent information about the research objective, confidentiality procedures, and their right to withdraw at any stage. Informed consent was obtained from all participants orally. The interviews were conducted and recorded with their explicit permission and were then fully transcribed. All identifying information was removed, and coding was used to anonymize the individuals.

Each interview lasted approximately 45 min. The audio was recorded and fully transcribed. A total of 10 interviews were conducted, covering approximately 25 pages of data. We ensured data saturation by continuing interviews and data collection until no new themes or insights emerged, indicating that the data were rich enough to comprehensively address the research questions. This process was guided by the principles of theoretical saturation (Corbin and Strauss, 2014), which emphasizes the point at which additional data no longer contribute to the development of new categories or themes. To monitor saturation, we continuously reviewed coding results and emerging themes throughout the analysis process, ensuring that patterns were consistently observed across cases and confirmed by multiple data sources. This iterative approach helped capture a full spectrum of perspectives and contextual nuances in the sample.

### 3.4 Data analysis

After conducting interviews with the participants in this study and collecting data from the research topic, we used MAXQDA 2020 software and were guided by the thematic content analysis approach (Braun and Clarke, 2021). Thematic analysis is the data analysis approach of this research. It can be used to analyze both large and small datasets—from case studies with 1 to 2 participants (e.g., Cedervall and Åberg, 2010) to large interview studies with 60 or more participants (e.g., Mooney-Somers et al., 2008)—and for both homogeneous and heterogeneous samples. Generally, there is less emphasis on the number of samples needed at the outset and more emphasis on identifying the appropriate number during the research implementation process (Fugard and Potts, 2015; Boyatzis, 1998). As we began the interviews in the present study with a small number of participants, we continued until theoretical saturation was reached. In total, based on the proposed model by Clarke and Braun (2013) for medium-sized projects like the present study, where the necessary data is collected from interviews, they suggested a number between 6 and 15 participants. In the present study, 10 participants were selected using a combination of purposive and snowball sampling. In similar studies, this sample size has also been shown to be sufficient. Such as Anderson et al. (2023) and Hoffkling et al. (2017). Additionally, similar to the research by Annosi et al. (2025), a purposive sampling strategy was adopted to meet specific criteria, ensure, and consequently increase the relevance and transferability of the findings. The criteria included research background, work experience, education, focus on sustainability, and willingness to participate.

First, In this study, the data was cleaned and organized into plain text, and all responses were stored digitally. Then the interview transcripts were carefully read, and the initial coding tags for all statements were coded to identify the initial coding codes. After removing duplicate data and merging initial codes with similar concepts, organizing themes were formed, and similar organizing codes were grouped, leading to the identification of overarching themes (Braun and Clarke, 2006).

To ensure reliability based on specific qualitative research criteria, inter-coder reliability was used. To calculate reliability in this way, the second author of this study was asked to randomly select and code the data obtained from the interviews for a second time as a research collaborator. Intra-rater agreement percentage, which serves as an indicator of analysis reliability, was calculated. The inter-coder reliability for the interviews conducted in this study was found to be 82.7%, which, considering that this level of reliability is greater than 60%, confirms the trustworthiness of the coding (Lincoln and Guba, 1985).


$$\begin{array}{l} \text{Inter}-\text{rater Agreement Percentage}\\ =(2\times \text{Number of Agreements}/\text{Total Number of Codes})\times 100\%. \end{array}$$

To ensure the validity of the qualitative section of the research, the technique of comparing evidence with existing literature was also used. To ensure that the concepts were systematically interconnected and internally consistent, multiple sources, evidence, rich descriptions of the dataset, and the definition of the research boundaries were utilized. Additionally, using member checking techniques and analyzing the coded data and results, five interviews were returned to the interviewees for confirmation and correction, all of which were confirmed, and their suggested points were considered (Lincoln and Guba, 1985).

## 4 Results

Data analysis in the present study was conducted using thematic analysis. After the interviews with the participants in this study were conducted, the interviews were transcribed. All texts were imported into the MAXQDA 2020 software, and all statements were coded for the identification of basic codes. After removing duplicate data and merging base codes with the same concepts, organizing themes were formed. Finally, based on the framework of core human resource management processes, similar organizational codes were placed together, and overarching themes were identified.

The first interviewee, in response to the question of how effective artificial intelligence can be in attracting and hiring talented individuals, stated:

"Artificial intelligence can significantly speed up the screening process by scanning resumes and identifying qualified candidates. Additionally, AI-based video interviews can help assess candidates' skills, such as communication and problem-solving. So, AI can automate work processes and automatically perform repetitive tasks. This increases productivity and frees up time for employees, allowing them to focus more on strategic and creative tasks. And it can even help organizations identify, develop, and retain top talent."

The appropriate base codes for this sentence (resume screening, video interviews, talent management, process automation) were used.

The fourth interviewee stated in response to the same question:

"Intelligent decision-making systems evaluate employees without human conflicts or discrimination and based on specific rules and criteria. This leads to fair, reasonable, and unbiased decision-making. Furthermore, intelligent decision-making systems are capable of continuously improving the selection process by utilizing intelligent algorithms and evaluation criteria. Intelligent algorithms are capable of analyzing the past and predicting future employee performance in various situations."

The appropriate base codes for this sentence (Elimination of conflicts and bias, use of intelligent algorithms) were used.

The first part of the human resource management processes is related to recruitment and hiring. The interviewees’ responses were analyzed line by line, and the extracted codes are shown in Table 5.

**Table 5:** Basic and organizing themes related to artificial intelligence in intelligent recruitment and hiring.

Extracted themes	
Basic themes	Organizing themes
Resume screening (5 times), Elimination of conflicts and bias (4 times), Workforce strategic planning, job requirements analysis, automated resume analysis (2 times), resume analysis, filtering, and speeding up the recruitment process (3 times).	Resume screening
talent management (4 times), use of intelligent algorithms (6 times), process automation (3 times), Recruitment data analysis, smart hiring, talent identification, skills-needs matching, skills gap analysis (2 times), candidate performance evaluation, smart selection, skills and experience assessment (3 times)	Intelligent candidate assessment
video interviews (6 times), Reliance on artificial intelligence, accurate and rapid analysis (2 times), virtual interviews, video interviews, smart interviews	video interviews

The second interviewee, in response to the question of how artificial intelligence can help improve employee skills and performance, stated:

"AI-based tools can be very effective when combined with the philosophy of gamification in education, provided we have a specific scenario and a pre-defined goal. Using AI helps employees identify their training needs and provides customized training programs to improve performance. It can also assist both staff and managers in the employee performance evaluation process. By analyzing data and using intelligent algorithms, individual performance can be evaluated transparently and based on accurate data, and solutions for improvement can also be provided. Additionally, it can be used for training and developing employee skills. By experiencing scenarios and interacting with AI models, employees can experience real-world conditions in simulated environments and improve their skills."

The appropriate base codes for this sentence (Intelligent training, Intelligent training needs assessment, Intelligent performance management and evaluation, Training and Development) were used.

The seventh interviewee stated in response to the same question:

"Using algorithms to create accurate employee profiles, including skills, experiences, strengths, weaknesses, and professional development needs, can be very helpful in managing and improving performance."

The appropriate base code for this sentence (employee profiling) was.

The second part of the human resource management processes relates to performance management. The interviewees’ responses were analyzed line by line, and the extracted codes are shown in Table 6.

**Table 6:** Extracted themes related to artificial intelligence in intelligent performance management.

Extracted themes	
Basic themes	Organizing themes
Intelligent performance management and evaluation (5 times), Performance evaluation, performance prediction, and capability (2 times), eliminating human conflicts and discrimination (3 times), pattern prediction, performance prediction system	Intelligent performance analysis
employee profiling (6 times), Ethics and Privacy Issues (2 times), Humanity and Human Values, Immediate Feedback (4 times), Personalized and Customized Feedback	Employee profiling
Intelligent training needs assessment (4 times), Training and Development (4 times), process automation (2 times), Goal setting, development planning (2 times)	Intelligent career development planning

The third part of the human resource management processes relates to human resource training and development. The interviewees’ responses were analyzed line by line, and the extracted codes are shown in Table 7.

**Table 7:** Extracted themes related to artificial intelligence in education and the intelligent development of human resources.

Extracted themes	
Basic themes	Organizing themes
Intelligent training needs assessment (3 times), employee profiling (6 times), needs forecasting (2 times), pattern forecasting.	Artificial intelligence in education and the intelligent development of human resources.
Intelligent training (4 times), Training and Development (4 times), Lifelong Learning	Providing intelligent educational content.
process automation (2 times), Reliance on artificial intelligence (2 times), training effectiveness	intelligent assessment of training effectiveness

The third interviewee, in response to the question of how AI can help create a fair and transparent system for compensation and benefits, said:

"AI-based systems can automatically detect and reward employee performance. It can also play a significant role in analyzing data for employee reward and recognition management, and by referencing performance and established criteria, it can determine rewards, promotions, and incentives fairly and based on the individual's added value."

The appropriate base codes for this sentence (Recognition and reward program; Reward and incentive management) were used.

The seventh interviewee stated in response to the same question:

"AI-based systems can be used by examining and evaluating an individual's past performance and creating fair and transparent reward and compensation systems."

The appropriate base code for this sentence (Pay transparency) was.

The fourth part of the human resource management processes relates to compensation and benefits. The interviewees’ responses were analyzed line by line, and the extracted codes are shown in Table 8.

**Table 8:** Extracted themes related to artificial intelligence in intelligent compensation.

Extracted themes	
Basic themes	Organizing themes
Reward and incentive management (5 times), Transparent communication with employees, using clear and fair criteria	Dynamic reward systems
process automation (7 times), Recognition and reward program (5 times), identifying top performance	Intelligent identification of top employees
Pay transparency (3 times), No conflict of interest, resource allocation based on capabilities and experience.	Personalized intelligent rewards

The third interviewee, in response to the question of how artificial intelligence can help create a happy and vibrant work environment, stated:

"AI-based systems can help employees maintain a happy and energetic presence in the workplace by providing relaxation exercises and stress management techniques. Also, based on each individual's needs, AI can suggest deep breathing exercises, meditation, or short fitness routines. Artificial intelligence can play a significant role in improving coordination and communication within teams and organizations. Using artificial intelligence algorithms can help predict needs and collaboration opportunities within an organization, facilitating improved communication and coordination. Therefore, this system can help create a happy and vibrant work environment."

The appropriate base codes for this sentence (Team coordination and communication, Stress and rest management) were used.

The ninth interviewee stated in response to the same question:

"Artificial intelligence can help organizations create an environment that supports innovation."

The appropriate base code for this sentence was (Innovation-oriented).

The fifth part of the human resource management processes relates to the retention and maintenance of human resources. The interviewees’ responses were analyzed line by line, and the extracted codes are shown in Table 9.

**Table 9:** Extracted themes related to artificial intelligence in intelligent preservation and maintenance.

Extracted themes	
Basic themes	Organizing themes
Team coordination and communication (6 times), Decision support, communication support, pattern forecasting	Intelligent prediction of employee behavior
Stress and rest management (2 times), Innovation-oriented (2 times), Effective communication, intelligent emotional recognition, supporting work-life balance (2 times), customizing the employee experience (3 times)	Enhancing employee experience
process automation (2 times), Collaboration and support, rapid adaptation, analysis and forecasting of requirements (2 times)	Intelligent forecasting and retention of talented employees

**Figure 1:**
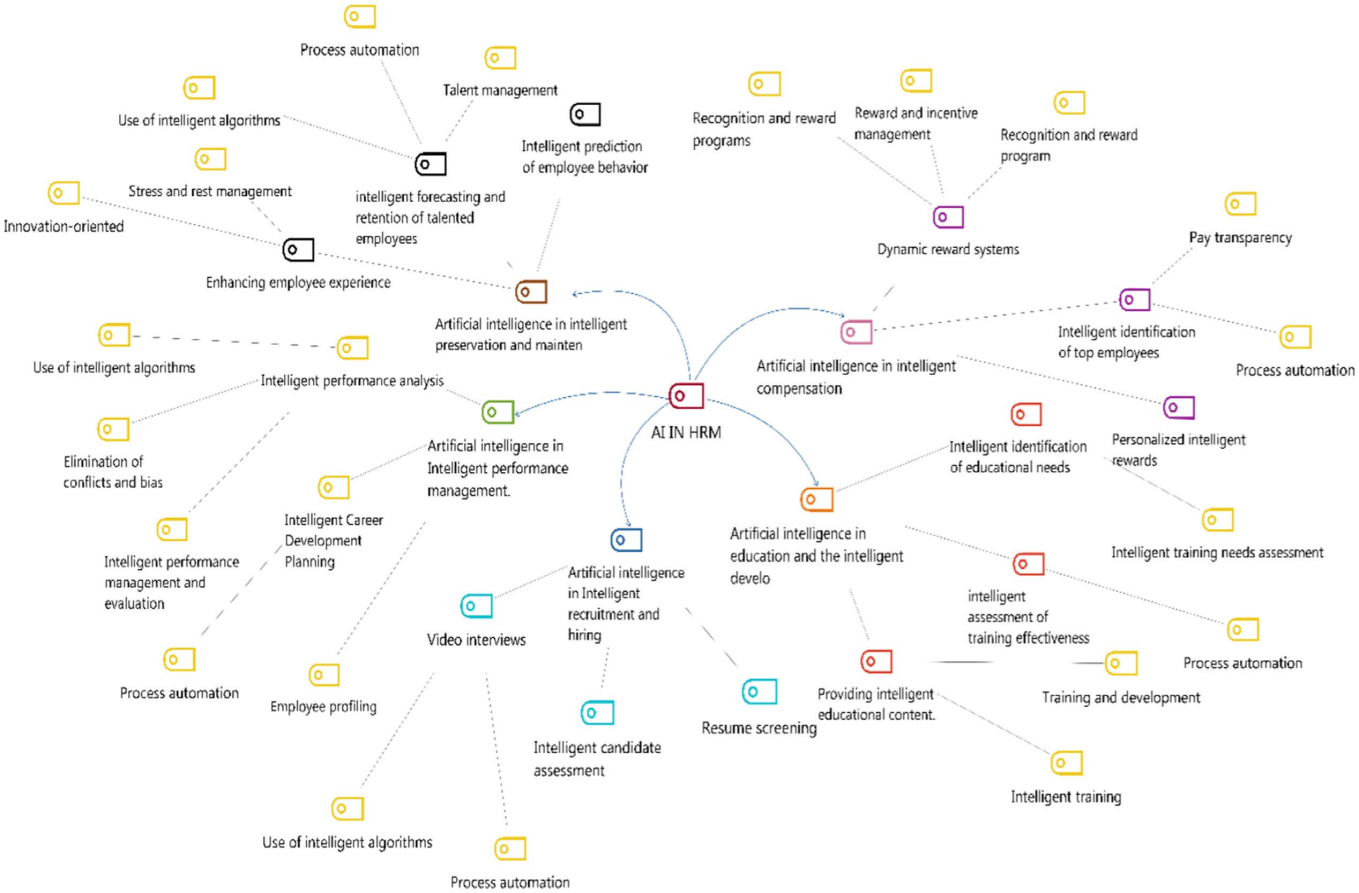
The final model for applying artificial intelligence in human resource management.

Finally, the final model for different human resource management systems is shown in Figure 1.

## 5 Discussion

Using artificial intelligence algorithms to automate and improve the recruitment and hiring process, from screening resumes to conducting initial interviews and intelligently evaluating candidates. According to the fifth interviewee, *“AI can significantly speed up the screening process by scanning resumes and identifying qualified candidates.”* Artificial intelligence allows you to find candidates with the skills and experience you are looking for using various filters, enabling you to connect with potential candidates and build a network of talented individuals. The third interviewee stated, *“Artificial intelligence can help organizations identify, develop, and retain top talent.”* Many repetitive tasks in the hiring process, such as sending emails and scheduling interviews, are automated. By providing accurate and understandable data, artificial intelligence helps you make better hiring decisions. With the help of AI and the use of video interviews, candidates can answer interview questions anytime, anywhere they wish. These interviews are automatically recorded and analyzed. It uses AI to analyze candidates’ body language, tone of voice, and other nonverbal cues to help assess their soft skills.

AI-based performance management systems use AI algorithms to evaluate and provide feedback to employees. These systems can improve the process of performance evaluation and improvement. Intelligent performance management helps organizations streamline their employee performance management processes and make smart, data-driven decisions. By utilizing AI-powered tools and platforms, organizations are able to translate their strategic goals into individual objectives, track employee progress, provide constructive feedback, and ultimately improve overall organizational performance. By setting clear and measurable goals for each employee and tracking their progress over time, along with providing continuous feedback and fostering a culture of positive feedback within the organization, encouraging employees to give and receive constructive feedback can contribute to personal development and help employees identify their strengths and weaknesses, as well as plan for professional growth. The eighth interviewee acknowledged, *“Artificial intelligence can help in the employee performance evaluation process.”* By analyzing data and using intelligent algorithms, individual performance can be evaluated transparently and based on accurate data, and solutions for improvement can be provided. Ultimately, this leads to increased employee engagement, improved communication between managers and employees, and a greater sense of belonging to the organization.

Based on the diagnosis and identification of each employee’s training needs, personalized training programs are provided for each employee using interactive AI-based training methods. The first interviewee stated, *“AI tools can be very effective when combined with the philosophy of gamification in education, provided we have a specific scenario and a pre-defined goal.”* Additionally, training is evaluated to continuously improve program effectiveness. By providing a self-service portal, HR processes are simplified and made more efficient, allowing employees to easily access the information and resources they need. Additionally, by automating many HR processes, the organization’s time and costs are reduced, enabling employees to focus on their core tasks. The tenth interviewee stated, *“Using AI helps employees identify their training needs and provide customized training programs.”* By offering online training and courses, organizations can enhance their employees’ skills and prepare them for future challenges. By using analytical tools, managers can analyze workforce data and make better decisions about hiring, training, and workforce development.

Using artificial intelligence to design and implement fair and motivating reward systems aims to identify and recognize top employees, while also providing incentives tailored to each employee’s needs and interests. Organizing rewards based on individual and team performance and achievements, along with identifying factors influencing employee satisfaction and motivation. Using cloud-based software, personal information, employment history, salaries and benefits, and employee training and skills can be recorded and maintained, and working hours, leave, missions, and other attendance-related events can also be tracked. The sixth interviewee stated, *“AI-based systems can automatically detect and reward employee performance.”* It also helps with calculating and paying employee wages, managing benefits and deductions, and preparing financial reports. On the other hand, the first interviewee stated, *“Artificial intelligence can play a significant role in managing employee rewards and recognition.”* By analyzing data and citing performance and established metrics, AI can fairly determine rewards, promotions, and incentives based on an individual’s added value.

Using machine learning algorithms to analyze past employee data and predict their future behaviors and performance can help human resources managers effectively forecast and manage employee behaviors and needs using this information. Using natural language processing to identify employee needs and problems is helpful for providing faster and better solutions to improve their experience. Predictive algorithms can also be used to identify talented and valuable employees and take measures to retain them.

Using artificial intelligence and analyzing employee activity data helps managers identify factors influencing satisfaction and improve the employee experience by examining work behaviors, increasing productivity, and maintaining a work-life balance. This feedback and evaluation help managers implement personnel-related improvement solutions. By using artificial intelligence and analyzing employee activity data, managers can identify factors influencing employee satisfaction and experience, and provide solutions for improvement in work processes and interactions with employees. Another interviewee explicitly stated that AI can help employees maintain a happy and energetic presence in the workplace by providing relaxation exercises and stress management techniques. Additionally, based on each individual’s needs, AI can suggest deep breathing exercises, meditation, or short fitness routines to help with better work-life balance planning and management.

## 6 Conclusion

Artificial intelligence is developing at an astonishing speed these days, and human resources departments around the world now consider it a necessity, not a luxury. Numerous AI-based tools, platforms, and applications are available to assist with everything from data-driven decision-making to task automation and employee engagement. Artificial intelligence, as long as it is human-made, can greatly simplify the hiring and recruitment process by reviewing resumes, identifying qualified candidates, scheduling interviews, and even automating initial interviews. Artificial intelligence can help employees upskill and improve their performance by providing personalized, data-driven training. By identifying the strengths and weaknesses of each employee, it can also provide suggestions for improvement. By analyzing performance data, including 360-degree feedback, productivity statistics, and performance evaluations, AI can provide employees with fair and reliable feedback. Identifying unconscious biases can also help achieve objectivity in performance reviews. The fourth interviewee stated “that data-driven AI operates based on specific algorithms and makes decisions impartially.” This can lead to the elimination of potential conflicts and discrimination based on gender, age, race, and other unfair factors in the decision-making and evaluation process.

Artificial intelligence, which is essentially a new tool in the field of education, along with its implementations, opens up new avenues for development and learning. The use of artificial intelligence in building and implementing these constructive personal development systems in relation to educational programs and personal development plans depends on processing individual performance data and their interests. Educational systems using artificial intelligence can provide personalized learning content, instant feedback, and unique guidance for each individual. The latest artificial intelligence technologies can be used to transfer knowledge and experience. The seventh interviewee stated “that artificial intelligence can be used for training and developing employee skills, and by experiencing scenarios and interacting with AI models, employees can experience real-world conditions in simulated environments and improve their skills.” Artificial intelligence can analyze large and complex datasets to identify important patterns, trends, and relationships, providing more accurate predictions for decision-making. AI systems can automatically search for and extract the necessary information and provide it to decision-makers.

AI tools can support managers in making strategic and informed decisions by meticulously analyzing data and providing research results. Artificial intelligence can help create a fair and transparent system for compensation and benefits by analyzing market-related data, such as salaries, benefits, and cost of living. It can get rid of issues related to relevant laws and regulations by observing anomalies in payroll data. Artificial intelligence, through data related to employee interactions such as surveys, feedback, and absence data analysis, can identify areas where employees need support to boost their morale and how to keep them engaged at work. It can also increase customer satisfaction by advising leaders on how to create a nurturing and supportive work environment. Artificial intelligence has redefined human resources by using machine learning algorithms that predict employee elements, such as predictive analysis of current employee data. The projected future growth is due to the use of natural language processing to identify employee needs and issues, which provides the context for better and faster solutions to their problems, leading to increased overall satisfaction. In this regard, the second interviewee stated that artificial intelligence can personalize the work experience by analyzing data and better understanding employees. For example, based on each staff member’s interests and needs, AI can provide them with relevant recommendations for projects, training, and morale-boosting activities.

Using predictive algorithms is a turning point in identifying and retaining talented and valuable employees. The truth is, if no action is taken to follow up with this group of employees, those involved in this process could damage their careers. Also, by using artificial intelligence and analyzing employee activity, managers can discover factors that impact employee satisfaction and experience, as well as find solutions for work processes and employee interaction. However, future research is invited to push the boundaries of knowledge. They may participate in validating our proposed models across various industries and sectors. They may also examine the impact of AI-based human resource management on organizational performance and organizational ethics, as well as employee well-being.

### 6.1 Limitations

While the objectives and priorities of this research have been achieved, the researcher faced limitations and challenges in reaching the desired goals. For example, Time and location constraints were one of the limitations of this research. In addition, the lack of knowledge and expertise of individuals in artificial intelligence, the lack of access to AI experts, and the lack of access to information are the biggest limitations of this research, which caused this study to be limited in the data collection process. Also, the data available in HR might be incomplete, inaccurate, or disorganized, making detailed analysis difficult. Finding relevant and reliable data on current HR practices was very difficult. This is due to a lack of data-sharing culture as well as privacy considerations. Research findings may be influenced by cultural biases, which makes it difficult to generalize the results to other contexts.

### 6.2 Suggestions for future research

Here are a few suggestions for future research on AI and HR. As artificial intelligence technology continues to evolve, it will likely have a profound impact on all aspects of work. Future research is essential to understand the impact of AI on HR and to develop strategies for using AI to improve the workplace for everyone.

- Review the current and desired states using gap analysis.
- The Future of AI in HR: What will be the role of AI in HR in the next 5 to 10 years? Using the foresight method.

In addition to these suggestions, it is important to conduct AI research in HR within the broader social and organizational contexts. For example, how does artificial intelligence interact with other trends such as globalization, technological change, and the increasing focus on diversity, equity, and inclusion? Considering these key factors helps us fully understand the impact of AI on HR and use it in a way that benefits everyone.

## Data Availability

The raw data supporting the conclusions of this article will be made available by the authors, without undue reservation.
